# The dark side of stemness – the role of hematopoietic stem cells in development of blood malignancies

**DOI:** 10.3389/fonc.2024.1308709

**Published:** 2024-02-19

**Authors:** Jadwiga Filipek-Gorzała, Patrycja Kwiecińska, Agata Szade, Krzysztof Szade

**Affiliations:** ^1^ Laboratory of Stem Cell Biology, Faculty of Biochemistry, Biophysics and Biotechnology, Jagiellonian University, Krakow, Poland; ^2^ Department of Medical Biotechnology, Faculty of Biochemistry, Biophysics and Biotechnology, Jagiellonian University, Krakow, Poland; ^3^ Doctoral School of Exact and Natural Sciences, Jagiellonian University, Krakow, Poland

**Keywords:** hematopoietic stem cell, preleukemic state, clonal hematopoiesis, acute myeloid leukemia, chronic myeloid leukemia, acute lymphoblastic leukemia, chronic lymphocytic leukemia, mature cell neoplasm

## Abstract

Hematopoietic stem cells (HSCs) produce all blood cells throughout the life of the organism. However, the high self-renewal and longevity of HSCs predispose them to accumulate mutations. The acquired mutations drive preleukemic clonal hematopoiesis, which is frequent among elderly people. The preleukemic state, although often asymptomatic, increases the risk of blood cancers. Nevertheless, the direct role of preleukemic HSCs is well-evidenced in adult myeloid leukemia (AML), while their contribution to other hematopoietic malignancies remains less understood. Here, we review the evidence supporting the role of preleukemic HSCs in different types of blood cancers, as well as present the alternative models of malignant evolution. Finally, we discuss the clinical importance of preleukemic HSCs in choosing the therapeutic strategies and provide the perspective on further studies on biology of preleukemic HSCs.

## 1 The phenomenon of blood production

Hematopoietic stem cells (HSCs) reside at the apex of the “hematopoietic tree” hierarchy and produce all blood cells throughout lifespan of an organism (1). HSCs possess the unique potential for both multipotent differentiation and self-renewal (2). They reside in specialized microenvironment called bone marrow niche, which regulates and drives HSCs activity (3).

HSCs are like a “hit squad”– innumerous, but extremely specialized. It is estimated that in adult humans 4.4 – 21.5 × 10^4^ of HSCs actively contribute to white blood cell production at a given moment (4). For comparison, one microliter of blood physiologically contains 4 –10×10^3^ of white blood cells (WBCs) and 3.5–5.2 ×10^6^ of red blood cells (RBCs), what gives in total 2-6 × 10^10^ of WBCs and 2-3 × 10^13^ of RBCs, assuming that average blood volume in adult human is 5-6 liters (5). Overall, approximately 10^6^ of new blood cells arise in each second, all of them descending from HSCs (1). This implies very high turn-over of hematopoietic cells and requires precise biological mechanisms to control this dynamic system.

**Figure 1:**
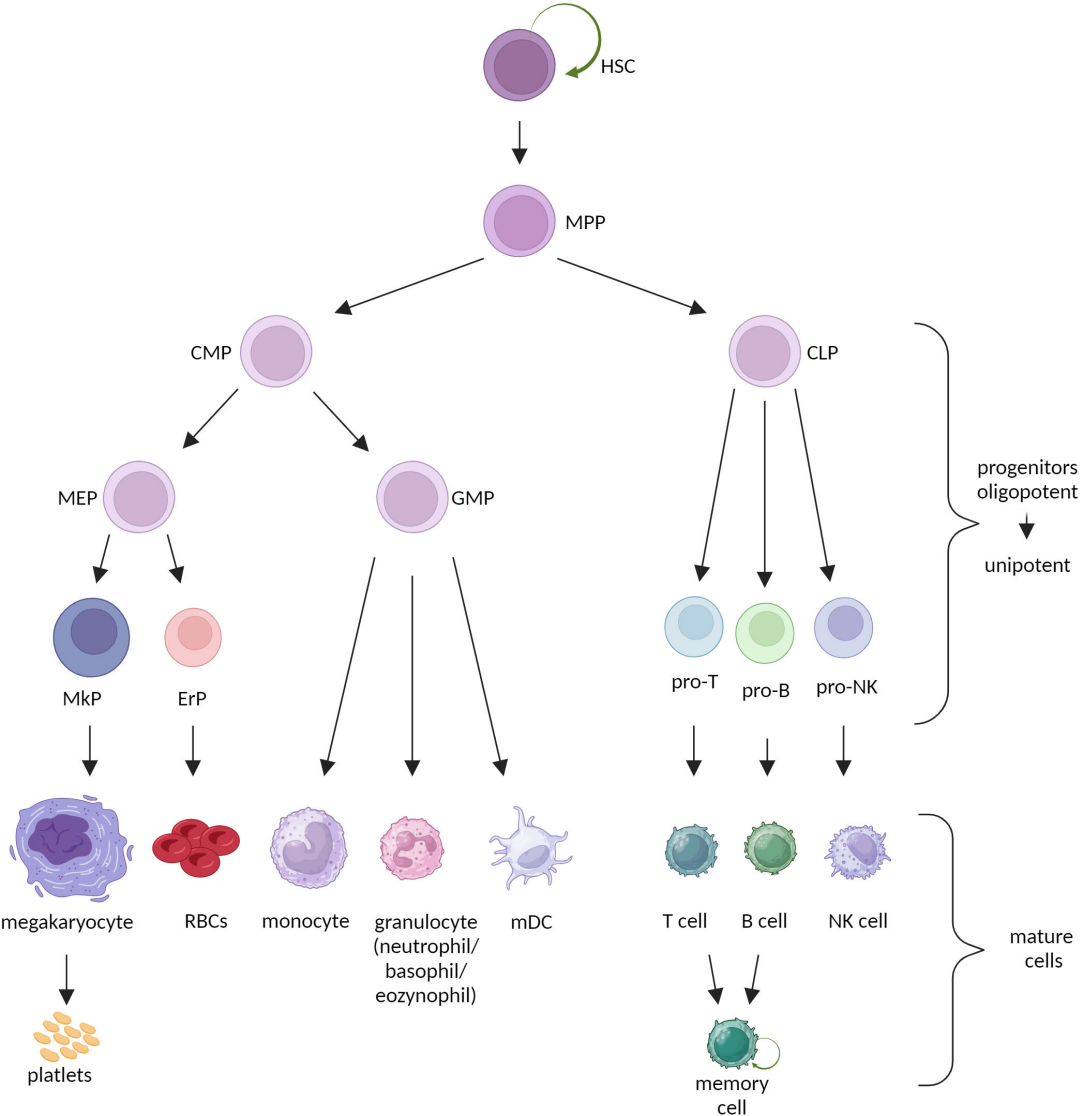
Classical model of hematopoiesis. HSC, hematopoietic stem cell; MPP, multipotent progenitor; CMP, common myeloid progenitor; CLP, common lymphoid progenitor; MEP, megakaryocyte-erythrocyte progenitor; GMP, granulocyte-monocyte precursor; MkP, megakaryocyte precursor; ErP, erythrocyte precursor; RBCs, red blood cells; mDC, myeloid dendritic cell.

HSCs undergo asymmetrical divisions to generate two daughter cells with different fates: 1) a stem cell that sustains HSCs’ pool, 2) a progenitor cell destined for proliferation and differentiation (6). This process is critical for hematopoietic homeostasis, as it maintains massive amplification of terminally differentiated cells of all blood lineages. However, stem cells can adjust their trend of division to a given situation and divide symmetrically, generating only stem or only progenitor cells (7, 8). Classically, HSCs give rise directly to the multipotent progenitors (MPPs). MPPs still maintain full lineage differentiation potential but lose the ability to self-renew. Downstream progenitors acquire lineage-specific potential, toward myeloid (common myeloid progenitor, CMP) or lymphoid (common lymphoid progenitor, CLP) lineage and gradually become oligo-, bi- and unipotent. Mature cells are the final step in hematopoietic differentiation. All populations of mature cells have relatively short lifespan [with only some exceptions like memory lymphocytes (9)] and perform highly specialized functions. Overall, maturation of HSCs entails increased proliferation capacity with decreased multipotency and self-renewal (**Figure 1**) (1).

However, the hematopoietic hierarchy in the classical model seems to be oversimplified and studies still define new hematopoietic stem and progenitor cell (HSPC) subpopulations, with mixed levels of stem and lineage-potential properties (10). Although by definition all HSCs are multipotent and can reconstitute whole hematopoietic system, analyses of human and murine hematopoiesis confirm that phenotypical HSCs population is intrinsically heterogeneous (11–15). First, detailed multicolor flow cytometry allows to establish set of markers that precisely distinguish multipotent progenitors from self-renewing HSCs, e.g. HSCs lack CD244 and CD48 (16, 17) but express CD150 (16) and EPCR (18). Next studies on mice divide the HSC pool into fractions based on the expression of cell surface markers: vWF (19), CD41 (20), CD61 (21), CD150^high^ (22), Neogenin-1 (Neo-1) (23), levels of c-Kit (24), or combination of CD49b and CD229 (25).

Each of these defined fractions preferentially differentiates toward selected lineage probably due to the transcriptional lineage priming (26). Nonetheless, the hierarchical relationship between the balanced and lineage-biased fractions within the HSC pool still remains not fully understood (27). The differences are also evident between HSCs from young and old individuals. Aged HSCs show limited self-renewal, lower regenerative potential and increased lineage-biased differentiation upon transplantation (22, 23, 27, 28).

The progenitor pool is also highly heterogenic. MPPs can be divided into four subsets (MPP1, MPP2, MPP3 and MPP4) according to lineage-bias, immunophenotype, molecular and functional characteristics (29). What is more, Yamamoto et al. described in mice myeloid-restricted repopulating progenitors (MyRPs) with high self-renewal activity – the population that falls outside the common scheme of hematopoiesis (30).

While all the recently described markers allow to distinguish new populations, there are also concepts suggesting that differentiation is not a stepwise but rather a continuous process (31, 32). The progenitor cell passes through lineage-committed transcriptomic states in a constant manner, without any halts between subsequent hematopoiesis levels, defined as cells with uniform phenotype and potential (32). Then the classification of cells to particular fractions would be rather indicative.

## 2 Hematopoiesis under pathological conditions

Blood is characterized by rapid cell turnover (33), multilevel hierarchical organization (even 17 to 30 maturation levels) (33), and relatively quiescent state of stem cells (2 to 20 months between cell divisions in humans) (4). A trade-off between limited divisional load (thereby reduced mutation rate) and continued descending toward the terminally differentiated and non-proliferating cells (with final “washing out” of cells, including cells with mutations, from the tissue) reduces accumulation of genetic alterations (34–36).

Despite mechanisms reducing excessive somatic evolution, blood malignancies accounts for more than 7% of cancer deaths (35, 36). Due to long lifespan and self-renewal, HSCs are especially prone to accumulate a set of mutations, what may initiate the clonal evolution toward hematologic malignancies (37). Although most of spontaneously occurring somatic mutations do not have a noticeable clinical effect (38) or altered cells are quickly removed by the immune system (39, 40), in some cases mutations can affect key genetic factors and provide a selective advantage to a particular cell, leading to its clonal growth (38). If the mutation happens at the level of non-stem cell, it probably disappears with the host cell due to physiological course of differentiation and cell death (41, 42). Thereby, HSCs with their natural self-renewing ability are suitable cellular target for primary malignant lesions (43). When HSC acquires first mutations, it turns into preleukemic HSC (44–46).

Large scale DNA sequencing of blood cells from people with no clinical signs of any hematological diseases revealed that this preleukemic state is relatively common and clearly age-related. It showed that among healthy adults, 1% below 40 years old and even more than 10% above 60 years old, already have clones of blood cells produced by preleukemic HSCs. This phenomenon is called clonal hematopoiesis of indeterminate potential (CHIP) (47, 48). The majority of CHIP cases are associated with mutations in *DNMT3A, TET2*, and *ASXL1* genes (49).

The strong association of clonal hematopoiesis with age and defined set of driver mutations projects the possible mechanisms of selection pressure. It was shown that hematopoietic clones with somatic mutations undergo both negative and positive selection (50). However, during aging of the organism the evolutionary paths based on only negative selection decline and the role of positive selection increases (50). This can be ascribed to the functional effects of the most common mutations in CHIP. The most commonly mutated genes - *DNMT3A, TET2*, and *ASXL1* – are epigenetic regulators (51). These mutations cause hypo- or hyper- methylation or dysregulate histone modifications. As the result, the mutated HSCs have higher self-renewal and disturbed differentiation what leads to their clonal dominance (52–55). Additionally, the expansion of selected clones during aging may be linked to increased inflammatory signaling in elderly individuals. It was observed that aging microenvironment may resemble the state of mild chronic inflammation (56). These similarities led to “Inflamm-aging” model that proposes the inflammatory signaling as one of the main driving forces of developing clonality in aging hematopoietic system (56).

On the one hand, preleukemic HSC seems to retain multilineage differentiation potential with both myeloid and lymphoid lineages, as majority of people with CHIP remain free of hematological diseases. On the other, preleukemic HSC already harbors some of the leukemia-specific mutations, has an increased ability to survive, a competitive repopulation advantage over non-mutated HSC and can undergo further somatic evolution (45, 46, 57). With such features preleukemic HSC may become cell-of-origin for hematologic neoplasm (45). This results in significantly high risk of blood malignancies among people with CHIP (58).

Although preleukemic HSCs’ existence often remains asymptomatic, sometimes it already causes noticeable hematological symptoms such as low-risk myelodysplastic syndrome (MDS) (59). Preleukemic HSCs' progeny not only inherit the baggage of mutations, but can also be affected by genetic hit themselves. The final, neoplastic mutation occurs at the progenitor cell level (44). The combination of all mutations leads to adaptive self-renewal activation and/or block in differentiation (44). Fully transformed progenitor with acquired self-renewal capacity is called the leukemic stem cell (LSC) or leukemia propagating cell (60, 61) (see **Box 1** with the nomenclature note). LSC directly gives rise to bulk leukemic blasts and sustains their production (61). It is important to state, that the involvement of preleukemic HSCs in leukemogenesis is well evidenced in two types of leukemia: acute myeloid leukemia (AML) and chronic myeloid leukemia (CML) – both called as paradigmatic HSC-source diseases (62). However, the importance of preleukemic stage likely differs in AML and CML. The preleukemic stage, with stepwise accumulation of epigenetic mutations in HSC, is essential in AML development. In contrast, it is thought that CML may develop due to single *BCR*-*ABL* translocation hit, while the potential prior preleukemic set of mutations only modulates the severity of the CML progression (62). But interestingly, there are some observations suggesting that HSCs may also contribute to generation of other types of hematopoietic neoplasms, including acute lymphoblastic leukemia (ALL) (63), chronic lymphocytic leukemia (CLL) (64) or even mature cell malignances (lymphomas) (65).

Box 1 **Nomenclature note:**

In the literature there is no consensus about precise definitions of discussed terms: cell-of-origin, cancer-initiating cell, cancer-propagating cells, cancer stem cell. In the context of leukemia, the terms take the form of cell-of-origin of leukemia, leukemia-initiating cell, leukemia-propagating cell, leukemia stem cell. These terms are very often interchangeably used, what might be misleading and cause ambiguous interpretations. To avoid misunderstanding, in this review we stick to the definitions described below.

The cell-of-origin of leukemia, or leukemia-initiating cell, is a cell that has the first leukemia-associated mutation(s). Although the presence of cell-of-origin in organism may be asymptomatic or show only dysfunction without signs of invasive disease, the cell-of-origin is the first link in oncogenesis chain, which may result in overt malignancy.

In contrast, LSC is a cell after the final genetic hit, and complete malignant transformation. LSC aberrantly acquires self-renewal and multipotency properties. LSC directly fuels (propagates) the full-blown leukemia development and can reconstruct the tumor after transplantation. Leukemia-propagating cell is a synonym for the LSC.

For example, in a majority of adult AML, the first leukemia-promoting mutation occurs at the level of HSC, making the preleukemic HSC the cell-of-origin of cancer. In turn, LSC presents a phenotype of more differentiated progenitor cell, but acquires self-renewal property thanks to the final set of mutations (43, 60, 61).

**Figure 2:**
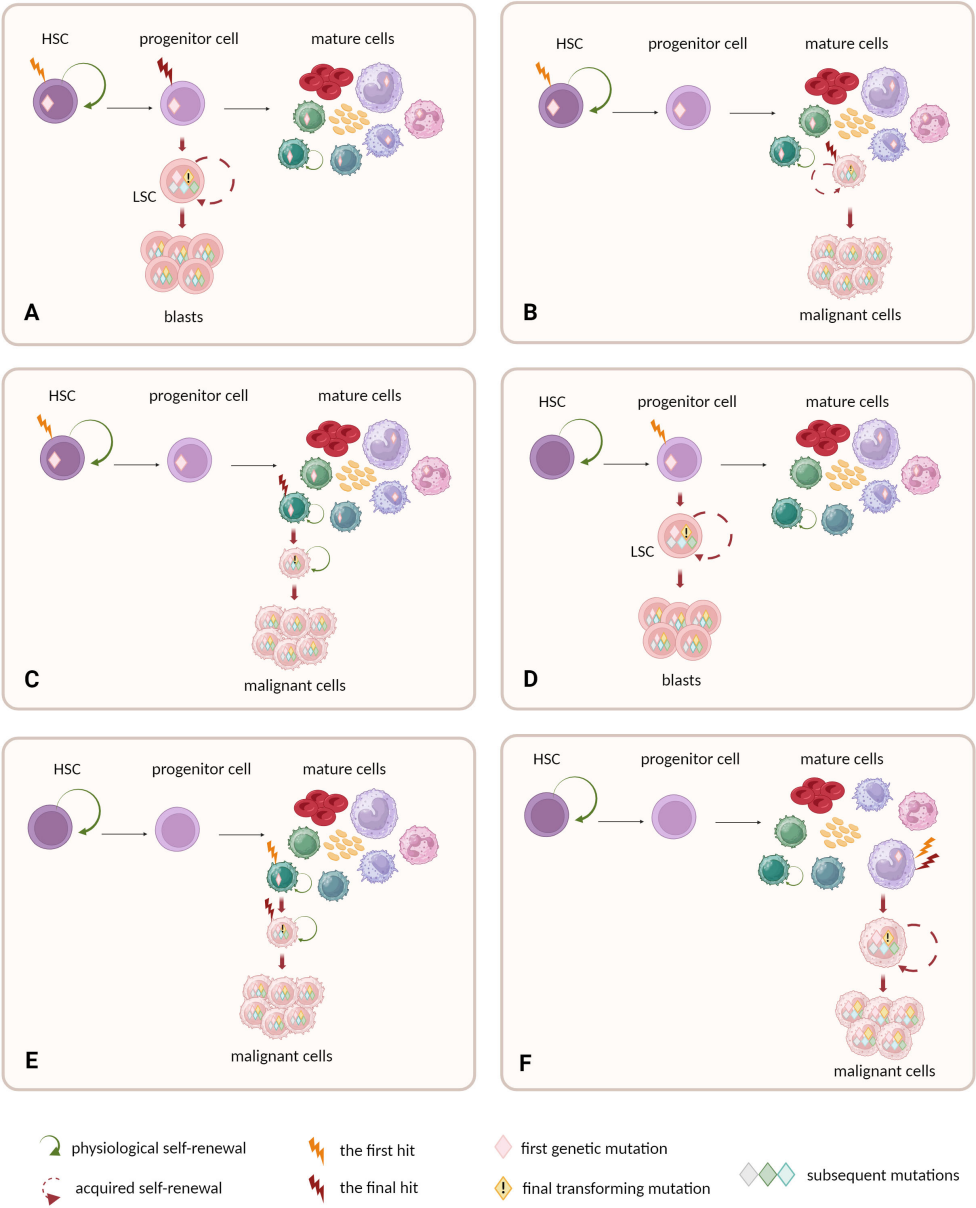
Possible cellular compartments for the first and final genetic hits. **(A–C)** HSCs are prone to accumulate preleukemic mutations, due to long lifespan and natural self-renewal properties. Progeny of mutated HSCs inherit the mutations. Final transforming hit may occur at different level of hematopoietic hierarchy: **(A)** in progenitor cell or **(B, C)** mature cells. **(C)** The natural self-renewal of memory B and T cells may further facilitate the accumulation of mutations and complete their malignant transformation. **(A–C)** The models assume that HSC constitutes the cell-of-origin, and progenitor or mature cell with self-renewal becomes the leukemia stem cell. **(D–F)** Other models do not ascribe direct role to HSCs in malignant transformation. **(D)** One possibility is that the early mutation warrants acquired self-renewal to progenitor cells that physiologically do not self-renew. This drives further accumulation of mutations and leads to malignant transformation. **(E)** Alternatively, the mature self-renewing cells like memory B and T cells may naturally accumulate mutations and constitute the origin of lymphomas or myeloma. **(F)** Non-self-renewing mature cell is unlikely to become the cell-of-origin of leukemia as mature cells are terminally differentiated. and have to short lifespan to accumulate mutations.

Nevertheless, apart from preleukemic HSC model that is demonstrated in AML and CML, there are other possible mechanisms among different levels of hematopoietic hierarchy that drive the origins of blood malignancies (**Figure 2**).

Although the self-renewal properties in adult organisms are mainly restricted to stem cells, there are still some exceptions such as memory B and memory T lymphocytes (9). These mature cells already have active self-renewal machinery and a relatively long lifetime, thereby are prone to accumulate mutations, similarly as in the case of HSCs (9). Moreover, the process of lymphoid cell maturation creates the opportunity to introduce malignant mutations. Genetic alterations during V(D)J rearrangement, class switch recombination or somatic hypermutation in B cells may activate oncogenes like *MYC* (66). Additionally, massive proliferation of lymphocytes can be induced by persistent antigenic stimulation during chronic inflammation, bacterial or viral infection and by signaling from self-antigens (66). Some B-cell neoplasms show phenotypic and genetic similarities with memory B cells, what might suggest that they are possibly derived from memory B cells (67). This group includes a subset of CLL with mutated IgV genes (68), hairy cell leukemia (HCL) (69), splenic marginal zone (SMZL) lymphoma (70), non-splenic marginal zone lymphoma (71) or mantle cell lymphoma (MCL) (72). Nevertheless, there are indications that HSCs contribute to development of at least some of lymphomas (65, 73–76).

Another possible scenario assumes reprogramming of primary non-stem cells into cells with *de novo* self-renewal properties. A set of acquired mutations can lead to aberrant activation of self-renewing machinery and then to leukemogenesis. Although the enforced self-renewal differs from intrinsic self-renewal potential of stem cells, it may be sufficient for tumorigenesis (77). Such exemplary mutation involves *KMT2A* gene (previously named *MLL*) (78). *KMT2A* can be rearranged by chromosomal translocation with one from at least 100 potential fusion partners (79). This aberration is observed in both acute myeloid and lymphoid leukemia patients (3% and 5% to 15% of cases, respectively) (79). It is also strongly associated with infant ALL (80). KMT2A fusion proteins probably promote uncontrolled self-renewal capacity in hematopoietic progenitors. Mouse model studies revealed that myeloid progenitors with *KMT2A* rearrangement express high levels of *HOXA9* – the gene responsible for self-renewal and immortality, normally expressed in c-kit-positive immature hematopoietic cells. Thus, *HOXA9* up-regulation can drive the acquisition of leukemic self-renewal activity among progenitors (81, 82). Next putative “offenders” are *MYC* mutations (78). *MYC* gene balances self-renewal and differentiation activity in HSCs (83). *MYC* deregulation is a frequent feature in B-cell lymphomas (84). Contrary to other lymphoma oncogenes, forced expression of *MYC* is sufficient to generate B-cell neoplasm in mouse model (85). Moreover, majority of murine lymphoma cells with *MYC* mutations seem to behave as cancer stem cells able to initiate and sustain tumor development (86). Other proposed examples of such oncogenic factors include mutations in *BMI1* gene and *MOZ-TIF2* fusions (78).

**Figure 3:**
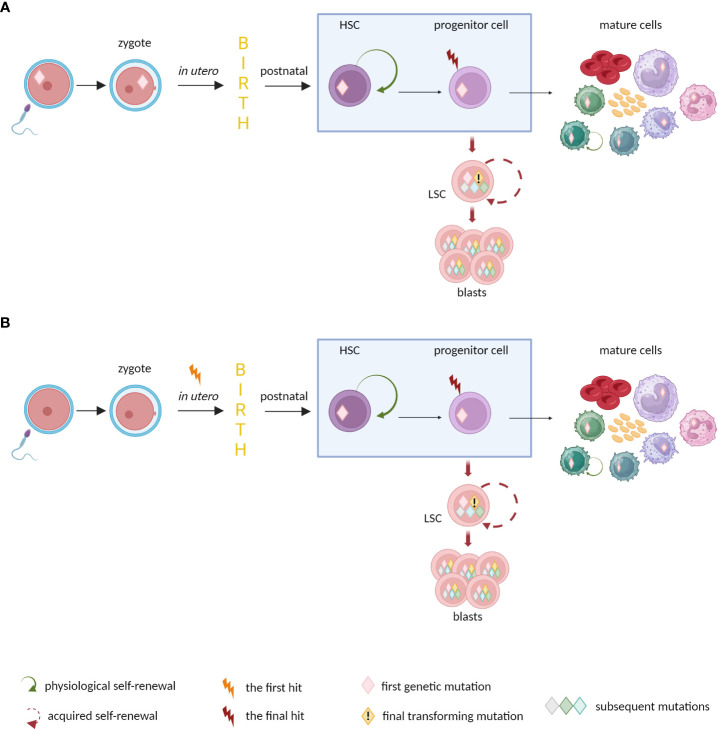
The origin of mutations predisposing to leukemia in newborns. **(A)** Germline and **(B)** *in utero* mutations may trigger preleukemic state and predispose to develop hematologic neoplasm. The disease may appear quickly after birth (e.g., infant ALLs), late in adulthood (e.g. MPNs), or do not occur at all, when no further somatic evolution happens.

Some children are born with mutations predisposing to leukemia (**Figure 3**). Since 2016, the World Health Organization Classification includes the entity called myeloid neoplasms with germline predisposition (87). The mutations are within the germ line, present in different cell types (not only in hematopoietic system), and can be handed-down. Individuals with germline mutations are at higher risk of developing myeloid neoplasms - especially MDS and AML. The germline mutations can act as first genetic hits in the process of disease development (88). Although myeloid neoplasms with germline predisposition are considered as rare, in many cases they probably remain underdiagnosed (89). Another option assumes *in utero* origin of leukemic mutations (63). These mutations happen in the fetus and are not inherited from parents. The prenatal mutation can initiate life-long clonal growth and evolution toward different hematologic malignancies, e.g. infant ALL (the most frequently *KMT2A-*rearranged) (90, 91) or myeloproliferative neoplasms (MPNs) in adult patients (92). Indirect evidence to support the prenatal origin of the first leukemic mutations comes from studies on twins. Monozygotic twins share the same early genetic lesions due to common hematopoietic system through vascular anastomoses. Dizygotic twins have entirely separate vascular systems so the mutational concordance is rather not observed (93).

Nevertheless, even if the morphology of neoplastic cells resembles more differentiated or mature cells, some recent studies indicate that first genetic hits might occur already at the stem cell level. The presence of the preleukemic states implies a question about mechanisms that drive the evolution of the premalignant clones and their transformation to overt leukemia. One of the commonly proposed triggers is an excessive immune response to common infections. Chronic inflammation is also able to initiate the clonal outgrowth of cells and activate preleukemic state, with possible further progression to overt leukemia (94). Furthermore, in case of childhood ALL, there is a hypothesis that the insufficient exposure to common pathogens during infancy may result in excessive and protracted immune response to later infections, what facilitates childhood ALL development (63, 95, 96). There are also reports about abnormal levels of pro-inflammatory cytokines in blood of infants, who later in life develop hematologic malignancies (97). Chronic inflammation is a characteristic feature of aging (98). The mutations in *DMT3A* or *TET2* - two most common epigenetic alterations observed in age-related clonal hematopoiesis and myeloid malignancies – initiate the proinflammatory state (99, 100). Other possible mechanisms include dysregulation of the endocrine system. Upregulated signaling from prolactin receptor creates pro-proliferative and antiapoptotic conditions and enhances resistance to cytarabine (101, 102). Recently increasing attention has been paid to the commensal microbes and their role in leukemia development. Studies in mouse models showed that disruption of microbiome by administration of antibiotics in young genetically predisposed mice was sufficient to promote transition from preleukemic state to overt B-ALL leukemia (103).

Importantly, the inflammatory conditions not only drive the occurrence of premalignant cells and facilitate their leukemic transformation, but also change their nearest microenvironment – the niche. Proinflammatory cytokines can transform the niche into leukemia-favoring area and fuel the genomic instability of hematopoietic cells (104, 105). Disturbance of only one factor in the niche can result in malignant transformation and development of hematologic neoplasm (3, 106–109).

The universal effects of the described excessive or prolonged inflammatory signals are elevation of ROS levels, shift from quiescent state to active cycling and high proliferation rate, what in turn enables evading from apoptotic pathways and exposes cells to higher risk of DNA damage (94). The source of genetic alterations may also originate in the natural processes responsible for development of adaptive immunity and recombination events. Recombination activating genes (RAG1 and RAG2) and activation-induced cytidine deaminase (AID) enzymes may contribute to chromosomal breakpoints and their translocations as a basis for further clonal evolution toward B-cell malignances (96, 110). Moreover, overactivation of AID may constitute a link between autoimmune diseases like lupus and increased frequency of lymphoid neoplasms (111).

Although the mentioned studies contribute to understanding the mechanisms beyond the malignant transformation, it is important to state, that it is often not clear whether the observed mechanisms act specifically at the level of stem cell or a premalignant clone.

Finally, the presence of premalignant state at the stem cell level prompts to review the current therapeutic strategies, such as autologous or allogenic hematopoietic stem cell transplantations. Therefore, the aim of this review is to determine the role of HSCs in the development of different blood malignancies in the light of current knowledge. We will investigate the process of tumorigenesis from the earliest stages and HSCs involvement in AML, CML, ALL, CLL as well as mature cell lymphomas.

## 3 AML – the paradigmatic HSC-source disease

AML is a hematological malignancy characterized by accumulation of abnormal blood cells with myeloid morphology. The disease predominantly occurs in later adulthood (median age at diagnosis is above 65 years) (112). Overall genomic landscape in AML is heterogenous and involves a wide spectrum of cytogenetic and molecular aberrations (113). Mutational profile has overriding impact on disease phenotype, course and outcome. Thus, classification of AML involves many different subtypes and it is still being complemented with new entities (114).

The concept of stem cell origin in cancer was discussed for years, but the detailed studies on AML provided one of the first elegant confirmations of this general notion. AML is the paradigmatic HSC-source disease with intrinsic cellular organization mimicking the hierarchy of normal hematopoiesis (115). Although LSCs in AML have the progenitor phenotype (116), the AML genesis begins within CD34^+^ CD38^-^ HSCs pool (45) (see **Box 2** for methodological aspects of assessing HSC biology). The full transformation to overt AML requires accumulation of a set of mutations in a single cell clone. Thus, HSCs with their natural long lifespan and self-renewal act as a refuge for accumulating mutations in the process of somatic evolution. The first somatic mutations turn self-renewing stem cell into preleukemic HSC. This stage is often clinically silent and remains undiagnosed. The final genetic hit occurs in progenitor derived from preleukemic HSC and transforms it into LSC. LSC acquires self-renewal properties, fuels disease progression and provokes its overt clinical manifestation (**Figure 2**). This general stepwise model is now well evidenced, and places preleukemic HSCs as evolutionary ancestors of frank AML development (44–46, 57, 123–125).

**BOX 2 Methodological aspect of assessing HSC biology.**

HSCs are commonly associated with Lin^-^CD34^+^CD38^-^ cell phenotype. But according to our best knowledge, flow cytometry gating based only on CD34 and CD38 markers is insufficient in studies on HSCs biology as downstream MPPs share the same phenotype and thus cannot be distinguished from HSCs (117). Functional analysis like clonal tracing (4, 118), FACS-sorted cell transplantations (119–121) or humanized mouse models (117, 122) reveal that hematopoietic stem cells with natural ability to both multipotent differentiation and long-term self-renewal are enriched in Lin^–^CD34^+^CD38^-^CD90^+^CD45RA^–^ fraction. We honestly believe that clear immunophenotypical distinction between self-renewing HSC and non-self-renewing MPP should be a critical aspect in such type of studies. However, some articles, also reviewed here, do not include Lin^-^CD34^+^CD38^-^CD90^+^CD45RA^-^ as the immunophenotype of highly purified human HSC, what may be the reason for inconsistent observations. Thus, interpretation of studies about HSCs should always be associated with a dose of criticism and analysis of applied methodology.

Additional level of complexity results from observations of intrinsic heterogeneity among human HSC pool (15), similarly as it is in murine hematopoiesis (23). In such case, bulk studies – although very valuable - are always restricted to average results across heterogeneous population. Therefore, analysis at the single cell level should be the essence of the projects about HSC biology, what may result in new clinically important outcomes.

Further studies revealed that the stepwise model in adult AML is also determined regarding the sequential order of mutations. First mutations appear in the “landscaping” genes, which regulate gene expression by epigenetic mechanisms, such as DNA methylation, histone modification or regulation of chromatin topology, e.g.: *DNMT3a*, *TET2*, and the members of the cohesin complex (45, 46, 57, 126–129). Some of the studies also rate *IDH1*, and *IDH2* genes as members of this group (46, 57). Mutations in the “landscaping” genes are often observed in preleukemic phase. Such aberrations can be detected in residual HSCs [Lin^−^CD34^+^CD38^−^CD90^+^, TIM3 and/or CD99 negative (130, 131)], myeloid blasts and non-leukemic cells from lymphoid lineage in samples from AML patients. Secondary hits involve genes belonging to proliferation signaling pathways, e.g. *FLT3* or *RAS* (45, 46, 57, 129). Those mutations are rarely found in residual HSCs, but are present in majority of blasts. Although AML blasts carry *FLT3*-internal tandem duplication (*FLT3*-ITD), the residual HSCs do not have this “late” mutation (45). Other classical example of gene, which is mutated at later stages, is *NPM1.* In *DNTM3a* and *NPM1* mutated AML, both *NPM1* and *DNMT3a* mutations were present only in CD45^dim^CD33^+^AML blasts, whereas HSCs/MPPs, lineage-committed progenitors, and CD33^−^mature cells harbored only *DNTM3a* mutation (57). The effect of late stage *NPM1* mutation among already mutated *DNTM3a* cells was further prospectively studied in mice (132). Mutation of *NPM1* in mice with *DNMT3a*-mutant clonal hematopoiesis (CH) caused progression to myeloproliferative disorders (132). In general, these “late” events are underrepresented during preleukemic stages, but directly precede the appearance of full-blown leukemia.

However, it is important to state that the “late” genetic events are relatively unstable between diagnosis and relapse, in contrast to rather stable presence of early mutations (46, 133, 134). The fact that “late” genetic events can be lost and/or substituted by another mutation implies that preleukemic HSCs may represent one of possible sources of relapse (46, 135). It is elegantly evidenced that preleukemic HSCs can survive standard induction chemotherapy and persist during remission (136, 137). In AML with *DNMT3a* and *NPM1* mutations at diagnosis, only the first one was detected during remission (57). Moreover, preleukemic HSCs retain non-leukemic reconstitution potential and actively give rise to multilineage mature cells. Lin^−^CD34^+^CD38^−^CD90^+^ preleukemic HSCs isolated from patients with A*ML1-ETO*-positive AML in long-term remission (up to 150 months) produced *AML1-ETO*-positive normal myeloid colonies but not leukemic blasts (138). Therefore, the early, “landscaping” mutations in preleukemic HSCs are stable between diagnosis and remission (46) or relapse (133), while induction therapy usually eradicates the clones with “late” genetic events (46). Importantly, the persistence of the preleukemic hits in *DNMT3a*, *SRSF2*, *TET2* and *ASXL1* during first remission is associated with unfavorable clinical outcome and higher cumulative risk of relapse (139). Overall, considering the intrinsic preleukemic HSCs’ features (like partial transformation or increased proliferation capacity) and further clonal evolution (enhanced by mutagenic chemotherapy), preleukemic HSCs should be considered as possible founders of new leukemic clones leading to relapse in AML.

The whole pathway from the first preleukemic hits to full-blown leukemia in adult AMLs usually takes years. However, it could vary significantly depending on the mutation profile. Few prospectively tracked patients with *DNMT3a, TP53*, spliceosome genes and *RUNX1* mutations at the time of the initial screening have developed AML within 7.4, 4.9, 6.7 and 1.5 years respectively (140). Additional data is delivered by studies on allogeneic bone marrow transplantations, after which both donor and recipient developed AML. This analysis indicates that preleukemic stage could take at least 7 years (141, 142). Other case report study shows that the delay can last even more than 15 years (143). Although these are rough estimations, they clearly indicate that adult AML development is usually a long-term process, spread over the years.

**Figure 4:**
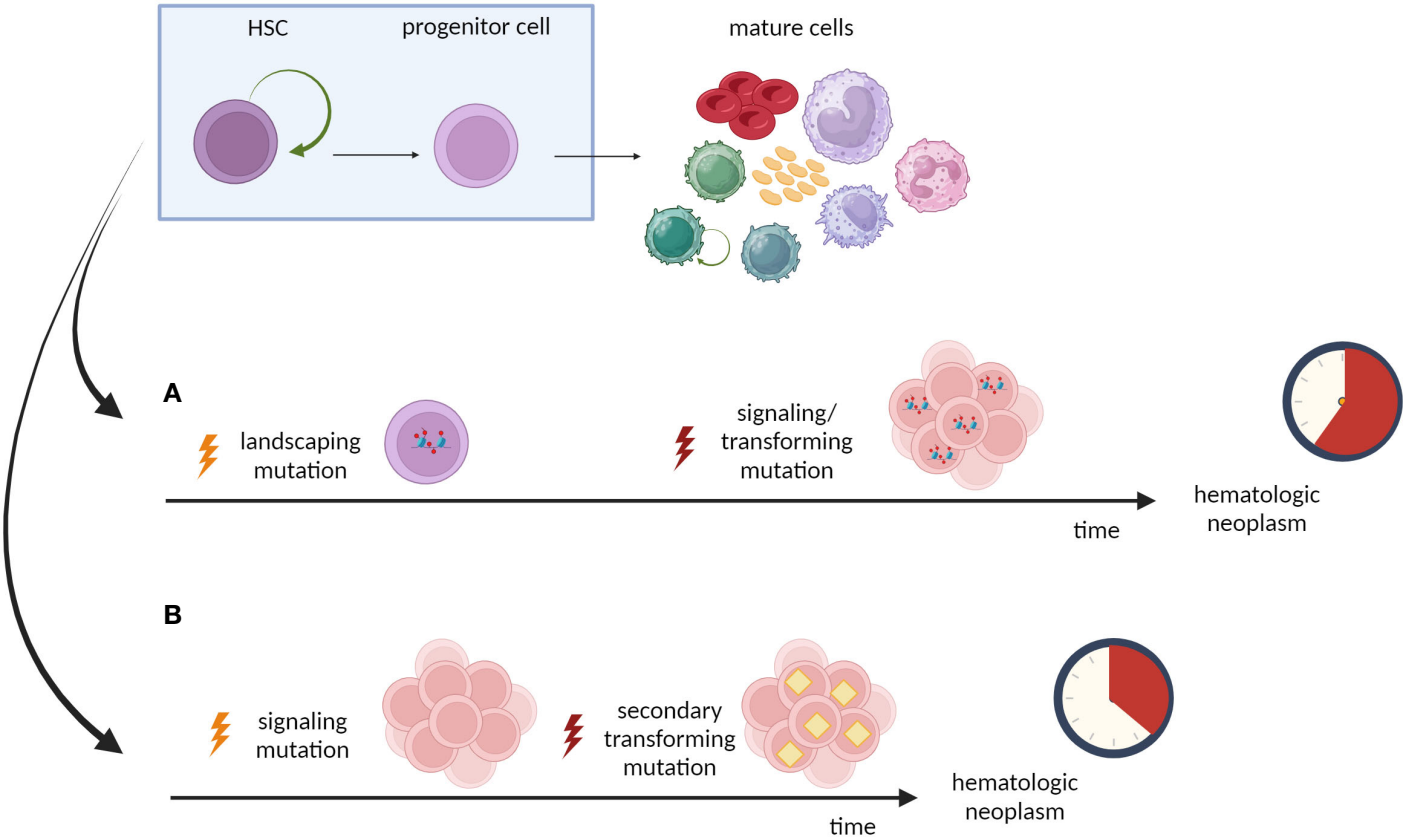
Effect of mutation order on leukemogenesis. Type of mutations and their order may determine the phenotype and the kinetics of neoplasm development. **(A)** The priority of the “landscaping” mutation (*TET2*) results in domination of *TET2* single-mutant cells within HSPC pool. The patients are older at the diagnosis, what indicates that acquisition of secondary/transforming hits requires more time. **(B)** The first hit in the signaling/proliferative mutation (*JAK2*) drives expansion of HSPCs pool and majority of these cells are *JAK2-TET2* double-mutant. Patients are younger at the diagnosis, indicating shorter time from first hit to overt neoplasm.

It is important to state that the length of preleukemic state is influenced by the order of mutation acquisition. It was elegantly evidenced in MPNs with *TET2* and *JAK2* mutations that type of mutations and their order determines the kinetics of neoplasm development (144, 145) The precedence of the “landscaping” mutation over the signaling/proliferative mutation (the scenario characteristic for AML) was associated with older age of patients than when the mutation order was reverse (**Figure 4**).

## 4 CML and its origin from HSC level

CML is a slow-growing myeloproliferative neoplasm, classically marked by the presence of the Philadelphia (Ph) chromosome – a product of a reciprocal translocation between chromosome 9 and 22, t(9;22)(q34;q11) (146). Resulting oncogenic *BCR-ABL1* fusion gene encodes a chimeric protein with constitutive tyrosine kinase activity (147). The disease predominantly occurs in late adulthood (148).

CML is the second paradigmatic HSC-derived hematologic neoplasm, however the preleukemic stage in CML has different character than during AML development (62). The CML hallmark - *BCR-ABL1* translocation - occurs at the level of HSC in almost all cases (149). Acquisition of such genetic alteration transforms a normal Lin^–^CD34^+^CD38^–^ HSC into a CML-initiating cell/leukemia stem cell, with a proliferative advantage and bias toward myeloid differentiation (62, 150). Importantly, natural self-renewal capacity of a primary cell, in which *BCR-ABL1* translocation occurs, is necessary for CML development as *BCR-ABL1* itself does not confer self-renewal (151–153). Some studies report detecting very low levels of *BCR-ABL1* transcript in peripheral blood from healthy individuals by sensitive RT-PCR methods (154–156). However, as these analyses were performed on whole peripheral blood, the readouts might come from cells other than HSCs, what explains a lack of clinical symptoms.

While the first genetic hit in CML is at the HSC level like in AML development, the initial accumulation of mutations in “landscaping” genes, typical for AML, is not always observed in CML. Minority of patients with CML (15-20%) have mutations in epigenetic regulators. Moreover, the order of the mutations is not determined like in AML. These “landscaping” mutations occur either before the Philadelphia translocation or are acquired during the treatment (after initial translocation occurred) (157, 158). This suggests that mutations in epigenetic regulators are not required for the CML manifestation. However, the order of their appearance is linked to the treatment outcome and progression to blast phase. Patients who have the “landscaping” mutations before the Philadelphia translocation respond to treatment better than patients who acquired new mutations during treatment (157).

Clonal somatic evolution of mutated HSCs may follow diverse paths in the course of CML. It is also possible that initial clones, present at low frequency at the beginning, expand during treatment. Indeed, both bulk and single cell analyses of *BCR-ABL1*-positive HSCs/LSCs revealed their functional diversity (159, 160). Identified subpopulations differentially responded to tyrosine kinase inhibitors (TKIs) treatment. Additionally, there was a subfraction of leukemic stem cells (CD45RA^−^c-kit^−^CD26^+^), which was quiescent and more resistant to therapy (160). The quiescent fraction of LSCs can reverse to active state and drive leukemia development (161, 162).

t(9;22)(q34;q11) translocation is presumably the necessary and sufficient condition to provoke clinical manifestation of CML, what may be explained by the wide spectrum of biological functions of the resulting *BCR-ABL1* fusion gene (163, 164). The *BCR-ABL1* fusion gene is recognized as a multifaced promotor of DNA mutations as the Ph-positive cells become more prone to accumulate genetic lesions (165, 166). The proposed mechanisms of its action are impairment of the DNA double-strand breaks repair (167) or augmenting the damaging impact of endogenous reactive oxygen species or genotoxic xenobiotics (168). Furthermore, *BCR-ABL1* may trigger DNA methylation changes and misshape the epigenetic landscape (169, 170). Thus, the Philadelphia translocation may be seen as the “landscaping” step in leukemogenic cascade, which facilitates further malignant transformation.

The *BCR-ABL1-*positive LSCs can drive further CML progression. Cooperation between increased level of t(9;22) transcript and genetic instability transforms relatively mild chronic phase to more aggressive and life-threatening blast phase. Expansion of BCR-ABL1 mRNA and protein is a hallmark of advanced stage (171, 172). Furthermore, Jamieson et al. reported that during blast phase Lin^−^CD34^+^CD38^+^ granulocyte-macrophage progenitors (GMPs), which present high levels of BCR-ABL1 and nuclear β-catenin, may turn into leukemic stem cells with self-renewal capacity (173). Therefore, LSCs in blast phase of CML can show different immunophenotypes: Lin^–^CD34^+^CD38^−^, Lin^–^CD34^+^CD38^+^ or even CD34^−^, like LSCs in AML (62). Secondary genetic lesions involve variety of changes – point mutations, gene deletions/insertions, chromosomal translocations or changes in number of chromosomes. Different mutations are found in myeloid or lymphoid forms of blast phase. The most frequent mutations occur within *TP53, RUNX1, CDKN2A/B or IKZF1* genes. Additional alterations in the *BCR-ABL1* kinase domain are also possible (174–179), but in general genetic and cellular heterogeneity in chronic phase of CML is much lower than observed in AML (62).

The discovery that Philadelphia translocation drives the CML and subsequent development of specific TKIs targeting the resulting fusion protein revolutionized hematology (180, 181). This achievement shows that is it possible to design a novel and specific clinical strategy targeting the source of malignancy. However, although the TKI therapy prolonged survival in many CML patients, CML LSCs may be insensitive to conventional treatment and persist during remission as a potential source of disease recurrence. Resistance can be primary or acquired due to subsequent *ABL1* kinase domain mutations (162, 182, 183). Despite achieving even long-term molecular remissions, only minority of CML patients can definitely discontinue TKIs treatment. Most patients need prolonged therapy, even throughout their life (184). TKIs inhibit LSCs proliferation, but do not induce effective apoptosis, especially among quiescent LSCs, as their survival seems to be BCR-ABL1 kinase independent (185–187). As total eradication of LSCs by conventional therapy is unachievable, alternative curative approaches involving kinase-independent intrinsic or cell-extrinsic mechanisms are still necessary.

## 5 HSC and acute lymphoid leukemia

As long as AML and CML are considered as paradigmatic HSC-source diseases, the role of HSCs in development of ALL is less clear. ALL is a heterogenous hematologic cancer, in which accumulated blast cells phenotypically resemble different stages of lymphoid progenitors’ differentiation. The disease is mainly diagnosed in children and young adults (incidence peak between age of 2 and 5 years), in a form of B-cell acute lymphoblastic leukemia (B-ALL) (>80% of ALL cases) (188). The most common chromosomal alterations in B-cell type are hyperdiploidy, *PAX5* alterations and recurring translocations: *KMT2A* rearrangements (v; 11q23); t(12;21)(p13;q22) encoding *ETV6-RUNX1*; t(9;22)(q34;q11) encoding *BCR-ABL1*; t(1;19)(q23;p13) encoding *TCF3-PBX1* (189).

Relevant observations about pediatric ALL come from retrospective analyses of neonatal blood spots from Guthrie Card and case studies on monozygotic twins with concordant acute lymphoblastic leukemia (63, 104). A few independent analyses of archived neonatal blood spots from hematooncological patients revealed that majority of them were positive for tumor-specific mutations at the time of birth (190–192). Thus, the first genetic aberrations predisposing to pediatric B-ALL seems to arise *in utero* (**Figure 3**). However, the prenatal event seem to be insufficient to cause overt leukemia (with some exceptions like *KMT2A* rearrangement). Monozygotic twins with concordant ALL can have different subsequent alterations (193–195). In addition, the latency period to clinical manifestation of leukemia can be variable. In a reported case, one of the twins with concordant ALL was diagnosed at the age of 14, 9 years later than the twin-sibling (196). Overall, disease development time can be prolonged up to 15 years after birth (197). Moreover, it is possible that only one twin from the pair develops leukemia, whereas second remains healthy. The concordance rate for disease development in twins ranges from at least 50% for children under one year old with *KMT2A*-r ALL to about 5–10% for other cases (104, 198). Concluding, these analyses coherently draw the two-step model of childhood ALL development. The first malignant transformation occurs prenatally and initiates a clinically silent pre-leukemic clone. To generate fully transformed cells, accumulation of secondary genetic lesions is necessary to complement the primary oncogenic event (199, 200). Fortunately, probability of transition from pre-leukemic to leukemic state in non-*KMT2A*-r ALLs is relatively low. It is estimated that almost 99% of *ETV6-RUNX1-*positive pre-leukemic clones never progress to clinical ALL (201).

Nevertheless, consensus about cellular origin of ALL is still elusive. According to current theory, particular genetic mutations, responsible for different types of B-ALL, occur at different levels of hematopoietic tree (202). Furthermore, in pediatric ALL stem cell hierarchy is disturbed, and phenotypically diverse blasts have stem-like properties. All leukemic subpopulations with CD34^+^CD19^-^, CD34^+^CD19^+^ or CD34^-^CD19^+^ immunophenotypes have the capacity to engraft and reconstitute the leukemia in immunodeficient mice (203, 204). This can be explained by the study on mouse model showing that B cells have unusually high degree of plasticity and under pathological condition, such as *Pax5* mutation, can dedifferentiate at least to the state of early lymphoid progenitors and produce functional T cells (205). Therefore, similarity between leukemia stem cell phenotype and given physiological B cell developmental stage is not necessarily indicative of the cellular origin of the leukemia (206).

Likely, the hematopoietic stem cell population is also altered, at least in some types of ALL. First, genes commonly mutated in ALL are involved in early lymphoid priming of hematopoietic stem and progenitor pool (207). Second, ALL recurrence is in majority caused by blasts arising from a clone ancestral to the leukemia at time of diagnosis (208). This builds rationale that HSCs are involved in ALL development. Till now, studies show that role of HSCs differs in various types of B-ALL with different etiological mechanisms.

### 5.1 ALL with *ETV6-RUNX1*

*ETV6-RUNX1* is the most frequent chromosomal alteration in ALL, related to almost 25% of pediatric B-ALL cases and probably exclusive for childhood. *ETV6-RUNX1* fusion gene (also known as *TEL-AML1*) is the product of t(12;21)(p13;q22) translocation (209). Fortunately, it is linked to a low-risk B-ALL and favorable treatment outcome. The translocation is a classic example of prenatal oncogenic event, occurring *in utero* and persisting postnatally (210–212).

Few independent studies using mouse models indicate that *ETV6-RUNX1* oncogene acts on the level of the HSCs, transforming them into pre-leukemic HSCs and cells-of-origin in ALL (213, 214). The induction of t(12;21) translocation at the pro-B-cell stage had no impact on B-cell development (200). Moreover, sequence analysis of *ETV6-RUNX1* breakpoints indicated that the translocations predominantly occur before B-lineage commitment, in cells lacking the expression of TdT or RAGs (110). Interestingly, acquisition of *ETV6-RUNX1* fusion does not cause any obvious phenotype alterations in HSCs. *ETV6-RUNX1-*positive pre-leukemic HSCs sustain normal hematopoiesis, quiescence, and small population size without growth advantage. Therefore, they can persist in the bone marrow and avoid exhaustion, but simultaneously accumulate subsequent genetic lesions (214, 215). *ETV6-RUNX1-*positive HSCs are not able to generate overt leukemia themselves, but require secondary genetic mutations (216). HSCs with the same *ETV6-RUNX1* breakpoints give rise to B-lineage committed progenitors with different immunoglobulin rearrangements (217, 218).

On the other hand, during full-blown leukemia, propagating stem cells are likely committed to the B-lymphoid lineage (202). Analysis of pediatric ALL samples revealed expanded CD34^+^CD38^−^ population (1.3% of all mononuclear cells (MNC); healthy subjects: 0.1–0.2%), consisting of two distinct subpopulations (1): major abnormal CD34^+^CD38^−^CD19^+^ cell subpopulation (1.1% of MNC) and minor CD34^+^CD38^−^CD19^−^ cell subpopulation (0.2% of MNC). In fluorescence *in situ* hybridization assay, all CD34^+^CD38^−^CD19^+^ cells but no CD34^+^CD38^−^CD19^−^ cells carried the *ETV6-RUNX1* fusion. In addition, all CD34^+^CD38^+^ progenitors were t(12;21) positive. Importantly, no CD34^+^CD33^+^CD19^−^ myeloid cells with the fusion gene were identified, indicating that *ETV6-RUNX1* fusion did not occur at multipotent stem cell level (202).

Another study described a case of twins – first twin was diagnosed with *ETV6-RUNX1-*positive B-ALL at age of 2 years while second twin remained healthy. The leukemic twin carried the CD34^+^CD38^−/low^CD19^+^ cancer-propagating population in bone marrow. Interestingly, the low number of CD34^+^CD38^−/low^CD19^+^ cells (0.002% of MNC) was also detected in the healthy twin, but not in hematologically normal age-matched children (219). The notable difference was that the CD34^+^CD38^−/low^CD19^+^ cells in leukemic twin expressed CD10, while the same population in healthy twin lacked CD10 expression. Thus, the CD34^+^CD38^−/low^CD19^+^ may act as preleukemic population. The question remains, what is the origin of this population: 1) does the *ETV6-RUNX1* fusion occur at HSC level and trigger the aberrant CD34^+^CD38^−^CD19^+^ phenotype or 2) the translocation arises in a B cell-committed progenitor that acquires this phenotype, while the CD34^+^CD38^−^CD19^−^ compartment retains normal in size and phenotype, without clonal involvement (202).

### 5.2 ALL with *KMT2A* rearrangements

Rearrangements of *KMT2A* gene are predominantly observed in infant acute leukemias and confer unfavorable prognosis (220). Despite advances in risk-adapted chemotherapy and novel therapeutic agents, the prognosis is still poor (221). In monozygotic twins concordance rate of developing ALL reaches even 100% (198). This aggressive form of leukemia likely results from biological properties of *KMT2A* fusion genes. This particular mutation is sufficient for leukemogenesis and possibly the process of malignant transformation is completed already *in utero* (63). Additionally, *KMT2A* rearrangements likely occur in CD34^+^ CD19^−^ cells (90, 222). Single-cell multiomic profiling reveals that in the peripheral blood of infant patients there is small population of cells that resemble HSPCs. This population presents CD34^+^CD19^−^ phenotype, express several canonical stem/progenitor tissue factors and is negative for B cell developmental genes. Simultaneously, some of these HSPC-like cells already carry *KMT2A* rearrangement and are able to generate leukemia in NSG mice (90). Nevertheless, we still lack the precise identification where at the hematopoietic hierarchy the *KMT2A* rearrangement occurs.

### 5.3 ALL with *BCR-ABL*

There are two types of t(9;22) translocations detected in some of the ALLs: one results in *BCR-ABLp210* fusion while the second in *BCR-ABLp190* fusion. The *BCR-ABLp210-*positive ALL likely represents another malignancy in which HSCs are the cells-of-origin (202). The fusion protein was observed in each analyzed stem/progenitor compartment from ALL samples: CD34^+^CD38^+^, CD34^+^CD38^−^CD19^+^, CD34^+^CD38^−^CD19^−^ as well as CD34^+^CD33^−^CD19^+^ pro-B cells or CD34^+^CD33^+^CD19^−^ and CD34^−^CD33^+^CD19^−^ myeloid populations (202). These data strongly suggests that *BCR-ABLp210* fusion occurs in the stem cell pool in this type of ALL. Nevertheless, *BCR-ABLp210* translocation is relatively rare in case of ALL, but is a classical hallmark of CML. Consistently, in both *BCR-ABLp210*-positive ALL and *BCR-ABLp210*-postive CML, multipotent HSCs are the cells of origin.

In contrast, t(9;22)-positive ALL with differently placed breakpoint encoding *BCR-ABLp190* fusion arises rather in B cell–committed progenitor cells as CD34^+^CD38^−^CD19^−^ population remains normal in size and along with myeloid cells are negative for *BCR-ABL1p190* fusion (202). The different target population of *BCR-ABL1p190* and *BCR-ABL1p210* hits reflects different biological activity of these two forms of fusion protein (223).

## 6 HSC and chronic lymphocytic leukemia

CLL is a lymphoproliferative neoplasm, characterized by an accumulation of clonal mature B cells with aberrant expression of CD5 and exclusively one type of immunoglobulin light chains (κ or λ). The leukemic cells infiltrate bone marrow, blood and lymph nodes. Presence of more than 5 × 10^3^/µL B cells in peripheral blood for at least 3 months is a clinical criterium for CLL diagnosis (224, 225). CLL could be divided in two groups based on somatic hypermutations within the variable regions of immunoglobulin heavy-chain (IGHV) in B-cell receptor (BCR). CLL subtype with mutated BCRs (IGHV-M) has favorable prognosis while CLL with unmutated BCRs (IGHV-UM) is linked to poor prognosis (226, 227).

Virtually every case of CLL is preceded by a pre-leukemic state called monoclonal B lymphocytosis (MBL) (228, 229). Patients with MBL have fewer than 5 × 10^3^/µL of circulating B cells, do not present disease-related symptoms, but the B cells are already monoclonal or oligoclonal. Patients with low-count MBL (<0.5x10^3^/µL B cells) are not expected to develop overt leukemia. Patients with high-count MBL (with 0.5-5x10^3^/µL of B cells) carry a 1–2% annual risk of progression to CLL (228–230). MBL is more frequent in older individuals (3 to 5% of general population over the age of 50 years) and can be detected up to 6 years before appearance of overt leukemia. In 20%–70% of cases MBL consists of more than one B cell clone (231). Similarly as in MBL state, in patients with CLL more than one clone can be detected in up to 14% of cases (232).

Apart of MBL state, CHIP also increases the risk of developing CLL. CHIP can be divided in two distinct groups: lymphoid (L-CH) and myeloid clonal hematopoiesis (M-CH) (233). The L-CH is linked to higher risk of CLL. However, L-CH is less frequent than M-CH, and L-CH-mutations occur at different levels of hematopoietic hierarchy (HSCs, immature lymphoid precursors, mature cells). In contrast to MBL, L-CH involves all clonal populations regardless of the status of IGH genes rearrangement (233).

The cellular origin of CLL is still unclear. Process of B cell maturation is complex and involves many different stages where the initial mutation may start the CLL leukemogenesis (234–238). One of the models assumes the involvement of HSPCs in CLL pathogenesis. Although this idea came up more than 20 years ago, the first retrospective analysis of CD34^+^ populations from CLL samples gave ambiguous results (239, 240). Genetic alterations in CD34^+^ cells were present only in some patients, but provided the proof of concept of possible early origin of CLL. The first study that prospectively evidenced an early origin of CLL from HSCs was presented by Kikushige et al. They observed that HSPC pool from bone marrow of CLL patients was normal in number and distribution of subpopulations (LT-HSCs, ST-HSCs, LMPP, MPP) and did not have rearranged IGH genes, presenting germline configuration. The number of B cell precursors (pro-B cells) (CD34^+^CD38^+^CD10^+^CD19^+^) was increased 5-times on average, but still presented polyclonal IGH rearrangement. In xenotransplantation assay, purified CD34^+^CD38^-^ HSCs from patients with CLL could both regenerate their own population and give rise to bilineage lympho-myeloid hematopoiesis, even after serial transplantation experiments. On the contrary, neither CD19^+^ CLL cells nor pro-B cells managed to make a stable engraftment. However, transplanted CD34^+^CD38^−^ HSCs frequently gave rise to CD19^+^ B cells with typical CLL-like phenotype, characterized by aberrant CD5 expression and mono- or oligoclonal IGH rearrangement. Interestingly, the IGH rearrangement in HSC-derived CLL-like cells was different from the rearrangement observed in CLL cells from the patient. Consistently, CLL-HSCs from single patient transplanted into several mice simultaneously gave rise to B-cells clones with distinct VDJ recombination (64). These results strongly support a role of HSCs in development of CLL (241).

Other strategy to verify the role of HSCs in CLL is based on analysis of typical for CLL genetic alterations among stem cell pool and cells from non-lymphoid lineages. Well-known chromosomal alterations in CLL are del13q14, del11q23, trisomy of 12 and del17p (242). Although del13q14 and del11q23 were detected in purified CD19^+^ CLL cells in some samples, none of them was detected among CD34^+^CD38^-^ HSCs or CD33^+^ myeloid cells from CLL patients (64). Additionally, CLL-HSCs transplanted into immunodeficient mice produced B cells without chromosomal alterations found in original CLL clone. The possible explanation is that these chromosomal alterations are not necessary for disease initiation and occur later in B cell development. On the other hand, CLL-HSCs showed high expression of early lymphoid transcription factors including *IKZF1* (*IKAROS*), *TCF3* (*E2A*) and *IRF8*, what likely caused the biased differentiation toward B cell lineage (64).

Collectively, the study by Kikushige et al. suggests that HSCs contribute to genesis of CLL. Although HSPC pool is not contaminated with detectable CLL clones, the HSCs from CLL patients already have biased differentiation toward B cell lineage. These HSCs drive expansion of polyclonal B cell progenitors. Next, auto- or xeno- antigens trigger BCR signaling, which expands mono- or oligoclonal B cell populations. This results in asymptomatic pre-leukemic state called MBL. MBL can further progress into clinical CLL due to additional chromosomal or genetic alterations (64, 241).

Next detailed studies based on mutation detection supported the direct role of HSC in CLL. Damm et al. showed that mutations in *NOTCH1, SF3B1, TP53* and *XPO1* genes were already present at the level of CD34^+^ multipotent hematopoietic progenitors in the majority of patients with CLL (243). Similarly, Quijada-Álamo et al. demonstrated that mutations in *NOTCH1, MYD88, FBXW7* and *XPO1* as well as chromosomal 11q and 13q deletions appeared in CD34^+^CD19^−^ hematopoietic progenitors (244). On the contrary, *TP53* and *SF3B1* mutations occurred later in maturation (244). Marsilio et al. analyzed different hematopoietic fractions: HSC plus MPP (HSC+MPP, Lin^−^CD34^+^CD38^low^CD45RA^−^CD90^+/−^); downstream hematopoietic progenitor cell populations containing CLP and CMP (Lin^−^CD34^+^CD38^+^); mature T cells (CD3^+^CD19^−^) and monocytes (CD14^+^CD19^−^) in samples from CLL patients. They demonstrated that in some cases the initial mutation occurred already in cells with HSC or MPP phenotype (245). Moreover, Agathangelidis et al. showed that ultra-stable CLL (lasting at least 10 years without progression), high-count MBL and low-count MBL samples had similar genomic profiles. Polymorphonuclear cells from the same CLL samples carried somatic mutations also present in MBL/CLL clones (246). Altogether, this supports the model of human CLL pathogenesis that begins with lymphoid clonal hematopoiesis driven by initial mutation at the stem cell level, through monoclonal B lymphocytosis, and ultimately overt CLL as a result of continuous malignant genetic evolution (247). Therefore, hematopoietic stem/progenitor cells can act as a cell-of-origin in CLL.

## 7 HSC and other mature malignancies

Role of HSCs in development of mature malignancies remains not fully understood. Nevertheless, several observations indicate the possible contribution of HSCs to some types of mature malignancies. S. Husby and K. Grønbæk proposed that initial genetic hit in mature lymphoid diseases may occur at three different levels: 1) immature or mature B/T cell, 2) progenitor cell or 3) stem cell level (65). Current evidence suggests that lymphomagenesis starts at stem cell level in at least some of the mature lymphoid malignancies, involving CLL (described in the chapter above), hairy cell lymphoma (HCL) and T-cell lymphoma (TCL) (65).

Majority of patients with HCL carry *BRAFV600E* gene mutation (248). Analysis of distinct subpopulations revealed that the *BRAFV600E* mutation could be found not only in HCL cells, but also among lymphoid progenitors (defined as CD34^+^CD38^high^CD10^+^CD19^+^), and HSCs (defined as CD34^+^CD38^−^CD90^+^CD45RA^−^) (73). However, the *BRAFV600E* mutation was much less frequent within HSCs in comparison to lymphoid progenitors, and no mutation was found among myeloid progenitors (defined as CD34^+^CD38^+^CD10^−^CD19^−^CD45RA^+/−^CD123^+/−^). Nevertheless, transplantation of highly purified HSCs from an untreated HCL patient to NSG mice gave rise to cells with HCL phenotype within 6 months, indicating the direct role of HSCs in HCL development. Consistently, mice with *BRAFV600E* expression in stem and progenitor cells (Mx-1-driven model) developed lethal hematological disease, in contrast to mice with *BRAFV600E* expression restricted to B cells (CD19-driven model), which did not present any hematological phenotype (73). Altogether, the *BRAFV600E* mutation likely occurs at HSCs level and initiates HCL development.

Next study investigated the role of HSCs in the development and treatment of multiple myeloma (MM). Sridharan et al. described six cases of patients with MM who underwent autologous hematopoietic cell transplantations (HCT) and later developed secondary AML/MDS (sAML/sMDS) (74). First, they showed that *TP53* and/or *RUNX1* mutations detected in sAML/sMDS, were already present in HSPCs at the time of transplantation (HCT was usually years before onset of sAML/sMDS). Second, in two cases the authors were able to sequence CD138^+^ myeloma cells at time of diagnosis and revealed that *TP53* mutation was the same in myeloma cells and AML blasts from sAML. The same mutations were also present in other lineages (T cells and granulocytes). Nevertheless, frequency of the particular mutations in HSCs was highly variable (from 0 to 55% VAFs), what might indicate different kinetics and schemes of clonal evolution between patients. These observations evidence that the mutated CD34^+^CD38^−^ stem/progenitor cells may contribute to both MM development and therapy outcomes.

The prospective studies on mouse models evidenced possible role of HSCs in development of B-lymphomas. One of the frequently mutated genes in lymphomas is a transcriptional regulator *CREBBP* (249). Horton et al. demonstrated that deletion of *CREBBP* in HSPCs (Mx-1 driven model) led to development of aggressive B-cell lymphomas (75). When *CREBBP* was removed in lymphoid committed progenitors (CD19-driven model), lymphoma occurred only occasionally and was much less aggressive. Moreover, authors checked whether the *CREBBP* mutations occurred at stem and progenitor level in 1 patient with diffuse large B-cell lymphoma (DLBCL) and 2 patients with follicular lymphoma (FL), without BM infiltration. The mutation was detected in one patient in myeloid colonies derived from CD34^+^CD38^+^ cells, but no mutation was present in colonies derived from CD34^+^CD38^−^ population. This may suggest that the *CREBBP* mutation occurs in HSPC pool (CD34^+^CD38^+^ phenotype), but not necessarily at stem cell level (CD34^+^CD38^−^ phenotype).

Other studies using mouse models investigated the development of mucosa-associated lymphoid tissue (MALT) lymphoma. The genetic hallmark of MALT lymphoma is a translocation of *MALT1* gene (250). The expression of human *MALT1* under the Sca-1 promotor in mice led to expansion of the HSPC pool. Murine Sca1^+^Lin^−^ immature cells with human *MALT1* expression had early priming toward B-cell differentiation and initiated clonal hematopoiesis that later progressed to MALT lymphoma. The malignant cells in mice reflected clinical, histopathological, genetic, and molecular characteristic of human MALT lymphomas (76). Furthermore, CD34^+^ cells from MALT lymphoma patients had more than 700 differentially expressed genes, in comparison to healthy controls. The most enriched pathways were linked to inflammatory response and antigen presentation. Thus, the pool of HSPCs may represent the origin of MALT lymphoma. However current data cannot point precisely whether MALT lymphoma starts at stem or progenitor level.

While some studies indicated the role of HSCs in some mature B-cell malignancies, other failed to find similar evidence. Molecular analysis on DLBCL or FL samples did not reveal CD34^+^ cells involvement in lymphomagenesis (251, 252). In another study on B-cell lymphomas, CD34^+^ cells were positive for CHIP-related mutations, but not for mutations frequently found in B-cell lymphomas (253). However, this may indicate that CHIP clones contribute to B-cell lymphomas, while investigated mutations occur at the level of more differentiated progenitors.

The mutational landscape of T-cell lymphomas also indicates possible common source with clones driving CHIP. *DMNT3A* and *TET2* mutations are frequently detected in CHIP and preleukemic phase of myeloid malignances (126). Interestingly, these mutations are also found in patients with T-cell lymphoid malignances. In T-cell lymphoma patients, the *DNMT3A* mutation was detected in both T-lymphoma cells and the CD19^+^ blood fraction, whereas the *TET2* mutation was shared between neoplastic clone, B cells and HSPCs (254, 255). The potential role of CHIP-related mutations in T-cell lymphomas might be important in the light of autologous chimeric antigen receptor (CAR) therapies. When patient’s own T-cells already harbor the *DMNT3A* or *TET2* mutations, the resulting CAR-T cells might be source of secondary CAR-positive T-lymphomas, however such cases seems to be very rare.

Mutations in *DNMT3A* and *TET2* genes can be also detected in classic Hodgkin lymphoma patients, but are rather infrequent (5 of 40 analyzed cases) (256). However, in some cases the malignant Hodgkin/Reed-Sternberg cells did not carry the CHIP mutations, which were frequent among other blood cells. This suggests that there is possible indirect role of CHIP in development of blood cancers. It is well evidenced that blood cells derived from CHIP clones show pro-inflammatory activity (257, 258). In turn, chronic inflammation may facilitate development of hematological malignancies (256). Thus, the discrimination between the direct (described as reservoir for driver mutations) and indirect, proinflammatory effect of clonal hematopoiesis is not obvious.

Tracing the direct involvement of CHIP-related HSCs clones to lymphomas is possible when MPN and lymphoma with common mutations occur in the same patient. In 90% of these cases time between MPN and lymphoma diagnoses is not longer than 5 years (averagely 1,5 years) (259). One study described 3 cases of angioimmunoblastic T-cell lymphoma (AITL) and concomitant neoplasm from myeloid lineage with common mutations (260). Moreover, one of those patients additionally developed genetically related DLBCL. The common mutations were mainly in *DNMT3A* and *TET2* genes, what builds rationale toward direct clonal hematopoiesis involvement in pathogenesis of those malignancies. Further evidence came from four patients with T-cell lymphomas of T follicular helper cell origin who later developed myeloproliferative neoplasms with the same CHIP-related mutations (261).

The mouse models also support the possible role of CHIP-related mutations in driving lymphomas. When *TET2* and *DNMT3A-*mutated HSPCs were transplanted into primary recipient mice, the animals developed AML or T-ALL. But after secondary and tertiary transplantation, the recipient mice predominantly developed lymphoma (T-cell angioimmunoblastic subtype) (262). This shows that stem cells with CHIP-related mutations may be the origin of T-cell lymphomas.

Other studies focused on cases with concurrent mantle cell lymphoma (MCL) and CML. MCL and CML probably do not share common cellular origin. The *BCR-ABL1* translocation in CML occurs at the stem cell level, but so far has not been detected in lymphoma clones (263, 264). Moreover, MCL is insensitive to specific TKI treatment against *BCR-ABL1* fusion protein (263). In these cases, the two diseases likely developed independently and do not have genetic relationship. Nevertheless, this observation does not rule out the possibility of stem cell origin in MCL (265). The alternative explanation is that MCL and CML originate from distinct HSC clones.

Finally, important observations came from autologous and allogenic HCT. The presence of CHIP in lymphoma patients at the time of autologous HCT is strongly associated with lower survival and increased risk of therapy-related myeloid neoplasm (266). There are also known cases of patients with MM who developed sAML/sMDS after autologous HCT (74). Next, few reports described the development of identical lymphoid malignancies in both recipients and donors after allogenic HCT (267–270). Molecular analysis revealed that in all cases the lymphomas were derived from donor cells, even if the donors did not have symptoms of malignancy at the time of transplantation. Moreover, the latency periods between allogenic HCT and diagnosis usually lasted several years and were similar in some of the donor-recipient pairs, what may indicate involvement of long-lived stem cells. However, in clinical settings the transplanted material usually consists of whole mononuclear bone marrow or mobilized blood, and stem cells constitute only minor part of the graft. Thus, it is possible that the transplanted lymphoma propagating cells were not necessarily a preleukemic stem cells. Transforming mutation might occur at different than stem cell level, but already warrants the self-renewal and drives the malignancy after transplantation. Nevertheless, given the increasing number of HCTs, including those for non-malignant hematological conditions, screening of the graft for known CHIP mutations might reduce the risk of graft-derived leukemia.

To sum up, several observations support the involvement of early precursor cells in genesis of at least some types of mature malignancies. The first mutations may originate in multipotent stem or progenitor cells and initiate the expansion of clone which has a potential to evolve into MPNs and/or lymphoid malignances after accumulation of subsequent mutations.

## 8 Perspectives and conclusions

The role of hematopoietic stem cells in hematologic malignancies is a subject of intensive research. As the HSCs’ involvement is virtually undoubted in AML and CML, there is also a growing body of evidence that stem cells contribute to at least some types of ALL, CLL and even mature cell lymphomas. If the assumptions about the preleukemic state of HSCs in different hematologic neoplasms are accurate, they carry relevant clinical implications.

An important issue is the discrimination between preleukemic HSCs and measurable residual disease (MRD) (127). MRD refers to leukemic cells resistant to chemotherapy and present in the patient’s sample during follow up. Positive MRD is a strong prognostic factor for subsequent relapse and shorter survival. Its detection has major impact on the decisions on further therapy (271). MRD analysis is mainly conducted by immunophenotyping or molecular testing (272). The choice of the most appropriate methodology for each case individually is a key point in obtaining reliable results. On the one hand, MRD testing using early genetic events like *DNMT3a* mutations can involve also preleukemic clones without overt transformation, giving falsely increased results (127). On the other hand, late events, which are absent in preleukemic cells, are significantly unstable during therapy (134). Thus, MRD monitoring using late events can lead to falsely decreased or even negative results, when the clonal evolution drives the expansion of leukemic cells with novel mutation. Nevertheless, even if preleukemic state does not define the disease itself or directly give raise to a leukemic clone, higher preleukemic burden is correlated with poor overall and relapse-free survival (137, 143).

**Figure 5:**
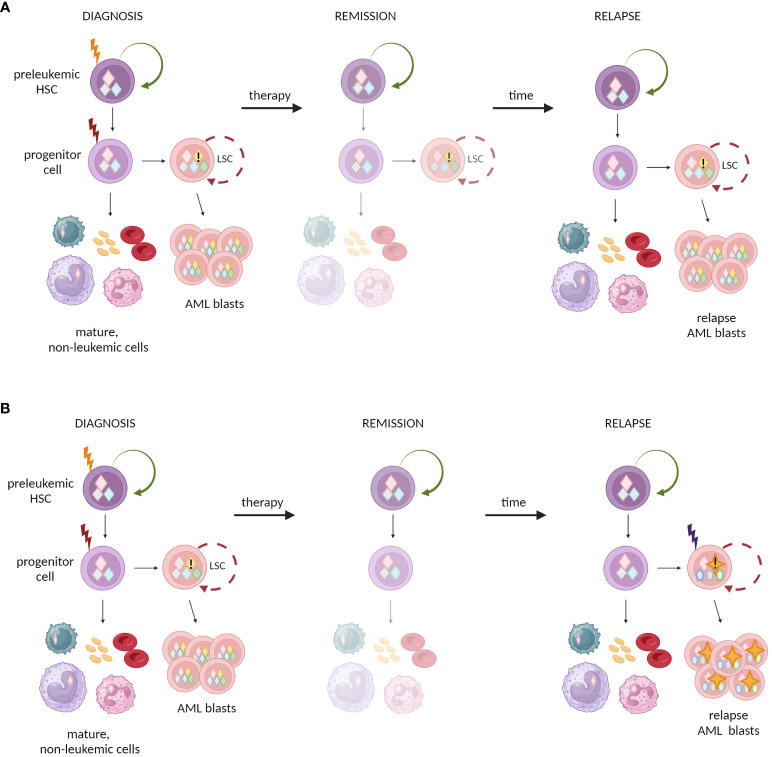
Possible mechanisms of relapse in AML after initial treatment and remission. **(A)** One possibility is that chemotherapy fails to eradicate LSCs that reconstitute the outburst of the leukemic blasts. **(B)** Alternative model assumes that chemotherapy eradicates the malignant LSCs, but causes new mutation in preleukemic clones, that in turn results in new clones of LSCs and leukemia relapse.

The crucial clinical question is whether preleukemic HSCs can drive leukemia relapse. One possibility is that the re-emergence of a disease is likely caused by incompletely eradicated cells, likely leukemia/malignant stem cells, from a primary dominant clone or its progeny. However, extensive genetic analysis of 8 AML patients indicated that relapse evolved from a minor subclone present at diagnosis (133). Thereby, it cannot be excluded that the new clones arise from preleukemic HSCs (**Figure 5**) (135). As it was elegantly evidenced, preleukemic HSCs are resistant to standard chemotherapy and can persist during remission (46). Induction therapy in AML patients leads to the expansion of non-leukemic hematopoietic clones, with typical preleukemic mutations (273). Moreover, current methods of therapy are strongly mutagenic themselves, so it is possible that preexisting preleukemic HSCs acquire additional genetic alterations and transform into fully leukemic cells (57).

The capacity of HSCs to restore the whole hematopoietic system is a basis of HCT (274). To underline the core role of HSCs in HCT, the procedure is often referred to as hematopoietic stem cell transplantation (HSCT). However, it is important to state that the term “HSCT” is misleading, as the functional HSCs represent a minor fraction of the transplanted material, and only in rare cases are enriched using the CD34 antigen (275). The importance of preleukemic HSCs is especially evident in the context of autologous HCT in the treatment of blood malignancies. Autologous HCT is predominantly used in MM (almost 50% of autologous HCT), non-Hodgkin lymphoma, or Hodgkin lymphoma (274). However, if the preleukemic HCSs contribute to different types of lymphomas and MM, the autologous HCT carries the risk of reinfusion of preleukemic HSCs that may drive the relapse or development of secondary neoplasm. Furthermore, even if HSCs carry a small set of asymptomatic genetic lesions, genotoxic treatment or conditioning facilitates further malignant transformation. This may contribute to neoplasm relapse after autologous HCT. Given that the typical preleukemic mutations occur in epigenetic factors, which regulate multilineage differentiation, the preleukemic HSC may drive therapy-related malignancies of different blood lineages (266).

However, allogenic transplantation can also be the source of preleukemic HSCs. There are many reports about so-called donor-derived leukemias or even co-occurrence of neoplasms in donor and recipient several years after allogenic HCT (276–278). Despite prior examination, the candidates for stem cell donors can asymptomatically carry abnormal HSCs, which would be harvested for transplantation and infused to the donor. Therefore, the detection of preleukemic mutations among donors for allogenic HCT would minimize the cases of donor-derived leukemias.

Finally, if the hypothesis about HSCs being a primary cells-of-origin in different hematologic neoplasms turns out to be true, it opens new perspectives for disease prevention. It is well evidenced that in adult neoplasms the period from the first oncogenic hit to clinical manifestation usually takes several years (127). This brings up the question whether it is possible to block the stream of somatic evolution and leukemia development at the early stages by detecting and eradicating preleukemic HSCs. Similarly, the presence of ALL-predisposing preleukemic HSCs could be checked during newborn blood spot screening and eradicated with targeted therapy.

It remains unclear to what extent the preleukemic cells can be removed by the immune system. The low mutation burden of preleukemic cells likely results in lower number of neoantigens, which can be recognized by T cells. Nevertheless, even in case of childhood leukemia, characterized by the low number of mutations in comparison to adult leukemias, the neoepitope landscape contains some possible targets (279, 280). It cannot be excluded that the preleukemic cells already have the immune evasive mechanisms allowing them to escape from recognition and elimination by the immune system, such as downregulation of antigen presentation, triggering co-inhibitory receptors on T cells (PD-1, TIM-3) or overexpression of ligands for anti-phagocytic receptors (CD47) on macrophages, which are typical for fully transformed leukemic cells (281).

The universal link between factors that contribute to development of premalignant state and further somatic evolution is the excessive or chronic inflammation. Understanding of those processes may possibly be used to prevent leukemic transformation. In case of childhood ALL there is a possible link between overactivation of immune response in case of insufficient exposure to common pathogens during infancy. This might provide rationale to propose non-specific “vaccines” for infants that would stimulate a mild, controlled immune response, which in turn would allow to avoid overactive, possibly leukemogenic, immune reactions during later childhood. We also know the common mutations related to CHIP. Thus, would it be possible to develop an anti-inflammatory therapy specific to the immune pathways related to these mutations? These are examples of how the recent research on premalignant states envisions new therapeutic possibilities.

**Figure 6:**
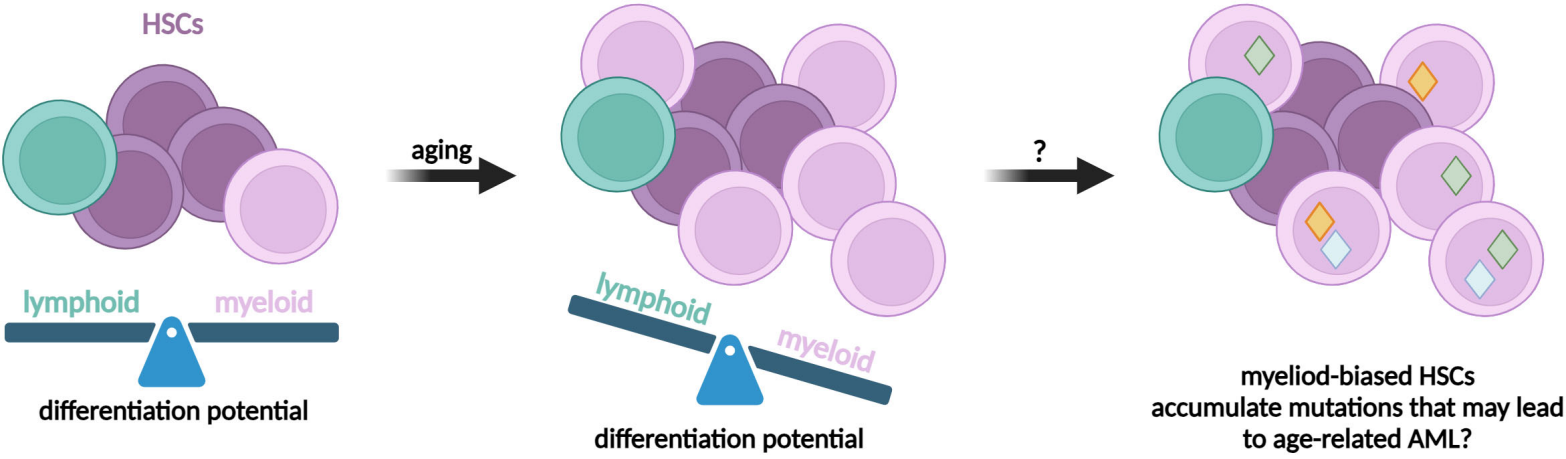
The potential role of lineage-biased HSCs in hematopoietic malignancies. Several studies evidenced the presence of self-renewing subpopulations of HSCs that are multipotent, but show preferential differentiation toward selected lineages, eg. myeloid. The lineage-biased HSCs expand during aging and therefore might be predisposed to accumulate mutations.

Finally, to avoid the risk of relapse or *de novo* blood neoplasms and to improve the safety of the HCT procedure, the novel strategies would have to prospectively differentiate preleukemic HSCs from non-mutated HSCs. But up to date, there are no markers specific for preleukemic HSCs (126). Nevertheless, some studies on mouse models propose a few markers to categorize a heterogenic pool of strictly defined murine HSCs (27). We and others showed that it is possible to prospectively isolate minor fractions of lineage-biased HSCs (23). The Neo-1^+^Hoxb5^+^ HSCs in mice show myeloid bias upon transplantation and a higher proliferation rate. Importantly, this fraction represents a minority in young individuals, but significantly expands during aging (23). The phenomenon of age-related myeloid-bias is observed also in humans (28, 282). While likely these myeloid-biased subpopulations are not mutated yet, it is tempting to hypothesize that they may be the origin of preleukemic HSCs, at least in myeloid malignancies (**Figure 6**). Additionally, there are other lineage-biased HSC fractions described (15). They may represent the source of preleukemic HSCs in case of other than myeloid malignancies or different age groups (see **Box 3** considering pediatric and adult leukemias).

Box 3 **Pediatric versus adult leukemias » same names ≠ same diseases.**

It is becoming more and more clear that pediatric and adult acute leukemias have significantly different biology. Studies by Dr. Meshinchi and his colleagues showed that the biological characteristics of AML are distinct in various age ranges and pediatric AML significantly differs when compared to adult AML (283). Adult AML is characterized by multiple mutations and alterations, accumulated over a long period of time. Leukemic cells from young patients predominantly carry structural alterations with a small number of DNA mutations. These few DNA alterations usually relate to specific genes, variants, hotspots or prevalence rates, different from that observed in adults (284, 285). Moreover, the genomic profile of childhood AML is very diverse as only a small subset of the mutations and structural alterations recurs within an observed group. That makes each case of pediatric AML more unique (283). Similarly, significant differences in gene profiles appear between pediatric and adult ALL (286, 287). These observations provide a rationale that pediatric and adult acute leukemias are distinct entities with different pathogeneses and treatment strategies.

Altogether, all of the mentioned possibilities require specific molecular or cellular markers, by which the abnormal cells can be prospectively isolated. We suspect that preleukemic HSCs present different gene expression profiles and functional properties than normal HSCs that not only increase their self-renewal, but affect their interactions with bone marrow niche, together leading to clonal advantage. Revealing these distinctive marks may facilitate compiling the protocols for distinguishing and targeting preleukemic HSCs. As preleukemic HSCs predominantly harbor mutations in epigenetic factors, epigenetic modification agents could be a promising tool to re-establish the physiology of the hematopoietic stem cell population. Nevertheless, all these mentioned possibilities require strict consideration in the context of real clinical usage, technical requirements as well as ethical and economic aspects.

To conclude, there is a growing amount of evidence indicating an active contribution of preleukemic HSCs to different hematologic malignancies. Preleukemic HSCs can be present not only in AML and CML – the paradigmatic HSC-source diseases, but may also be involved in other types of leukemia or tumors with mature cell phenotype. This exposes the possible next directions of clinical applications and opens new perspectives for disease prevention and treatment.

## Author contributions

JF-G: Visualization, Writing – original draft, Writing – review & editing. PK: Visualization, Writing – review & editing. AS: Writing – review & editing. KS: Conceptualization, Funding acquisition, Supervision, Writing – review & editing.
