# Correction: The dark side of stemness – the role of hematopoietic stem cells in development of blood malignancies

**DOI:** 10.3389/fonc.2025.1633832

**Published:** 2025-06-24

**Authors:** Jadwiga Filipek-Gorzała, Patrycja Kwiecińska, Agata Szade, Krzysztof Szade

**Affiliations:** ^1^ Laboratory of Stem Cell Biology, Faculty of Biochemistry, Biophysics and Biotechnology, Jagiellonian University, Krakow, Poland; ^2^ Department of Medical Biotechnology, Faculty of Biochemistry, Biophysics and Biotechnology, Jagiellonian University, Krakow, Poland; ^3^ Doctoral School of Exact and Natural Sciences, Jagiellonian University, Krakow, Poland

**Keywords:** hematopoietic stem cell, preleukemic state, clonal hematopoiesis, acute myeloid leukemia, chronic myeloid leukemia, acute lymphoblastic leukemia, chronic lymphocytic leukemia, mature cell neoplasm

In the published article, there was a mistake in the **Funding** statement. The grant number for the Narodowe Centrum Nauki Harmonia Grant was displayed as “2020/01/Y/NZ3/00121”. The correct statement is “The author(s) declare financial support was received for the research, authorship, and/or publication of this article. The work is supported by Narodowe Centrum Nauki Harmonia Grant nr 2018/30/M/NZ5/00869 and European Research Council Starting Grant “StemMemo” nr 101041737 granted to KS and by the Strategic Programme Excellence Initiative at Jagiellonian University.”

The authors apologize for this error and state that this does not change the scientific conclusions of the article in any way. The original article has been updated.

